# HNF1A-Maturity-Onset Diabetes of the Young (MODY) in a Family With a Rare Silent Variant: Clinical Challenges and Variable Expressivity

**DOI:** 10.7759/cureus.111076

**Published:** 2026-06-18

**Authors:** Rita Carvalho, Biana Moreira, Susana Parente

**Affiliations:** 1 Pediatrics, Unidade Local de Saúde da Arrábida, Setúbal, PRT

**Keywords:** hnf1a mutation, maturity onset diabetes of the young, monogenic diabetes, pediatric diabetes, synonymous variant

## Abstract

Maturity-onset diabetes of the young (MODY) is a monogenic form of diabetes characterized by autosomal dominant inheritance, typically occurring during adolescence or early adulthood. Mutations in the hepatocyte nuclear factor 1-α (HNF1A) gene are among the most common causes of MODY and are frequently misdiagnosed as type 1 (T1D) or type 2 diabetes (T2D), leading to inappropriate management. We report the case of a preschool-aged boy who presented with polyuria and polydipsia, fasting hyperglycemia, negative pancreatic autoantibodies, and an elevated C-peptide level. A multi-generational family history of diabetes was noted, and his older sister was previously diagnosed with T2D. Genetic testing using direct sequencing identified a rare heterozygous silent variant in exon 8 of the HNF1A gene (c.1533G>A; p. Gln511=) in the index patient, his sister, and his mother. To date, the mother remains entirely asymptomatic and normoglycemic. The variant is exceptionally rare in global population databases. Both siblings have maintained optimal glycemic control for four years through dietary modification alone, without requiring pharmacological therapy. This case demonstrates that rare silent HNF1A variants can be clinically pathogenic, likely by disrupting pre-mRNA splicing kinetics or mRNA stability. The observed familial segregation strongly suggests a clinical pattern of incomplete penetrance (an asymptomatic maternal carrier) and variable expressivity, potentially pointing to an attenuated, partial genetic defect rather than a complete loss of function. This case underscores the importance of integrating rigorous clinical phenotyping and familial segregation tracking alongside automated in silico predictions when evaluating synonymous variants in suspected monogenic diabetes.

## Introduction

Maturity-onset diabetes of the young (MODY) comprises a heterogeneous group of monogenic disorders caused by single-gene mutations with autosomal dominant inheritance, resulting primarily in pancreatic β-cell dysfunction [1-9]. MODY accounts for approximately 1-6% of pediatric diabetes cases [1,9]. To date, at least 14 genes have been implicated, with mutations in hepatocyte nuclear factor 1-α (HNF1A), glucokinase (GCK), hepatocyte nuclear factor-4 homeobox A (HNF4A), and hepatocyte nuclear factor-1 homeobox B (HNF1B) accounting for more than 95% of all cases. These subtypes differ significantly in prevalence, clinical phenotype, severity, associated complications, and therapeutic approaches [2,3]. Diagnostic challenges are frequent, with over 80% of patients initially misclassified as having type 1 (T1D) or type 2 diabetes (T2D) [5,9]. This report describes a family with MODY3 carrying a rare silent HNF1A variant, emphasizing the critical role of clinical phenotyping and family segregation in interpreting variants of uncertain significance (VUS).

## Case presentation

We describe the case of a previously healthy preschool-aged boy with a significant family history of diabetes. His maternal grandmother and aunt were treated with oral antidiabetic agents, whereas his paternal grandmother was on combined oral therapy with insulin. His sister had been diagnosed with T2D one year earlier during early adolescence. On examination, the patient’s height was at the 50th percentile, and body mass index (BMI) was 17.65 kg/m² (90th percentile). Over the preceding three months, he developed progressive polydipsia and polyuria. Home capillary glucose monitoring consistently showed fasting blood glucose levels above 126 mg/dL and postprandial levels exceeding 200 mg/dL. There were no clinical signs of insulin resistance, such as acanthosis nigricans.

Clinical and laboratory investigation

Laboratory evaluation showed normal blood gases, negative blood ketones, HbA1c of 5.3%, and an elevated C-peptide level of 4.5 ng/mL (reference range 0.8-4.2 ng/mL). Screening for pancreatic islet autoantibodies, including islet cell antibodies (ICA), glutamic acid decarboxylase antibodies (GAD), and insulin autoantibodies (IAA), was negative (Table 1). The absence of islet autoantibodies and preserved endogenous insulin secretion were inconsistent with the diagnosis of T1D [3,7,8]. Given the strong familial history of diabetes, the absence of severe obesity, and lack of clinical features of insulin resistance, an inherited early-onset form of diabetes was considered [3,7,8].

**Table 1:** Metabolic and immunological laboratory findings of the index patient and his sister Serum values at the time of initial clinical presentation are detailed. HbA1c: glycosylated hemoglobin; GAD: glutamic acid decarboxylase; IAA: insulin autoantibodies; ICA: islet cell antibodies

Laboratory Parameter	Index Patient (Brother)	Older Sister	Reference Range	Units
Fasting Plasma Glucose	146	137	60 - 100	mg/dL
HbA1c	5.3	5.7	4.27 - 6.07	%
Fasting C-peptide	4.5	3.2	0.8-4.2	ng/mL
Anti-GAD Antibodies	Negative (< 10)	Negative (< 10)	< 10	IU/mL
Insulin Autoantibodies (IAA)	Negative (< 20)	Negative (< 20)	< 20	IU/mL
Islet Cell Antibodies (ICA)	Negative (< 28)	Negative (< 28)	< 28	IU/mL
Total Cholesterol	122	155	< 170	mg/dL
Triglycerides	57	120	< 150	mg/dL

At the time of T2D diagnosis, the patient’s sister presented with polyphagia and obesity, but no acanthosis nigricans. Her metabolic evaluation revealed a fasting plasma glucose level of 137 mg/dL, HbA1c level of 5.7%, C-peptide of 3.2 ng/mL, and a normal lipid profile. The autoantibody test results were negative (Table 1). She was started on metformin (1000 mg/day) and achieved sustained HbA1c levels between 5.2% and 5.4%. On the basis of the clinical presentation and family history, MODY was suspected.

Genetic testing using polymerase chain reaction (PCR) and direct sequencing identified a heterozygous silent variant of HNF1A (c.1533G>A; p. Gln511=) (Table 2) in the patient, his mother, and his sister. Interestingly, the mother remained entirely asymptomatic, with no prior diagnosis of diabetes or history of hyperglycemia-related symptoms. The father tested negative for the variant. Other maternal relatives were not tested as they reside in a different country, with limited access to genetic studies, precluding further familial segregation analysis. This single-nucleotide variant is classified as a VUS according to the American College of Medical Genetics and Genomics (ACMG) criteria and the Rat Genome Database [10,11], whereas in silico analysis using MutationTaster predicts a pathogenic effect. This variant is exceptionally rare in the general population, with an allele frequency of 1.3 in 100,000 in exomes and 6.57 in 1,000,000 in genomes, as documented in population constraint databases [12].

**Table 2:** Molecular and genetic characteristics of the identified HNF1A gene variant Genomic coordinates and nomenclature are based on the human genome reference sequence. The classification follows the standard guidelines established by the American College of Medical Genetics and Genomics (ACMG). cDNA: complementary DNA; VUS: variants of uncertain significance

Gene	Chromosome	Exon	Nucleotide Change (cDNA)	Protein Effect	Classification (ACMG)
HNF1A	Chromosome 12	Exon 8	c.1533G>A	p. Gln511=	Variants of uncertain significance (VUS)

**Figure 1:**
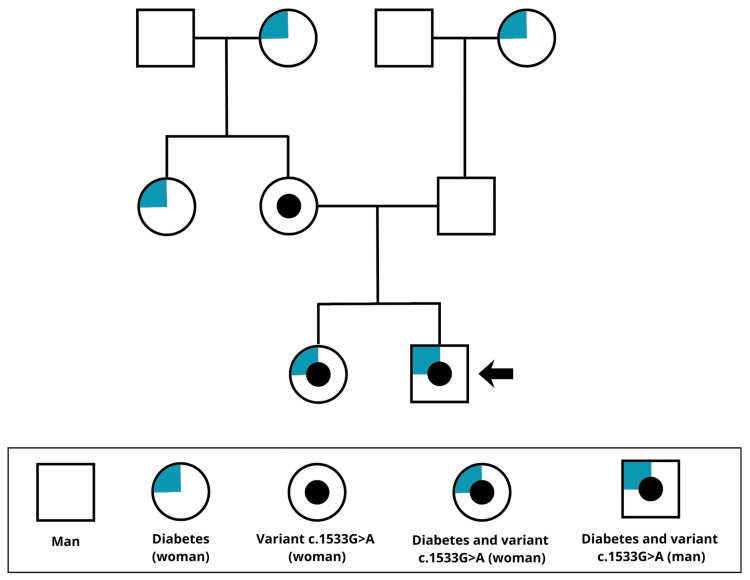
Multigenerational pedigree demonstrating HNF1A variant segregation Squares represent males and circles represent females. The proband (index patient) is indicated by the black arrow. Shaded blue quadrants represent a clinical diagnosis of diabetes, while central black dots indicate verified heterozygous carriers of the HNF1A variant (c.1533G>A). Unshaded symbols represent unaffected individuals. The figure (the familial pedigree chart) was designed and created using Canva (Canva Pty Ltd, Sydney, Australia).

The genetic segregation and clinical phenotypes of this family are illustrated in Figure 1.

Management and long-term follow-up

Dietary counselling with restriction of rapidly absorbable carbohydrates and encouragement of regular physical activity was implemented for the affected members. At the most recent follow-up, four years after the initial evaluation, the patient maintained an HbA1c of 5.1% and remained clinically well without pharmacological treatment. His sister also remained stable with an HbA1c of 5.6% after discontinuing medication two years after her initial diagnosis. Both siblings continue to be monitored biannually, reflecting the slow and mild progression of the disease in this specific family.

## Discussion

Mutations in the HNF1A gene account for approximately 52-65% of all MODY cases, making it the most common etiology of monogenic diabetes with autosomal dominant inheritance [1]. Affected individuals typically experience a progressive decline in pancreatic β-cell function, leading to the onset of hyperglycemia between childhood and early adulthood. Clinical features include osmotic symptoms (polyuria, polydipsia) or asymptomatic postprandial hyperglycemia (without ketosis or ketoacidosis). Key defining features of HNF1A-MODY include a strong multi-generational family history of diabetes, young age of onset, absence of insulin resistance, glycosuria, preserved endogenous insulin secretion, and the absence of pancreatic islet autoantibodies [1,5,7,8]. Despite these distinct characteristics, misdiagnosis remains remarkably common, with over 80% of cases initially misclassified as T1D or T2D, which frequently results in unnecessary insulin therapy or inappropriate management [3,4,6]. Diagnostic challenges arise frequently from the clinical overlap with polygenic diabetes, significant genetic heterogeneity, and limited clinical awareness among healthcare providers [1,3,6]. Consequently, MODY should be highly suspected in individuals presenting with young-onset diabetes who also have an affected parent, particularly when typical features of T1D or T2D are absent [6-8].

Clinical diagnostic criteria and screening strategies

To standardize recognition, current clinical diagnostic criteria for HNF1A-MODY emphasize a multi-generational family history of diabetes affecting at least two generations, a young age of onset typically before 25 years, and incomplete insulin dependency outside the normal three-year honeymoon period [6-8]. Additionally, classic criteria include the absence of pancreatic islet autoantibodies, the absence of typical T2D characteristics such as insulin resistance, acanthosis nigricans, or obesity, and the presence of low-threshold glycosuria at blood glucose levels below 180 mg/dL [6-8]. Genetic testing, particularly through next-generation sequencing, plays a central role in confirming the molecular diagnosis, guiding targeted therapeutic choices, facilitating family screening, and ultimately improving the cost-effectiveness of long-term care [1,5,7,8].

Differential diagnosis in early-onset diabetes

Distinguishing monogenic diabetes from polygenic forms represents a major clinical challenge, particularly given the rising global prevalence of childhood obesity [3,8]. In the present case, the initial classification of the patient's sister as having T2D was a logical consequence of her presentation with obesity and polyphagia. However, several clinical "red flags" raised doubts about this diagnosis and prompted further genetic investigation. The complete absence of clinical features of insulin resistance, such as acanthosis nigricans, alongside completely normal lipid profiles in both siblings, argued strongly against typical T2D [3,7,8]. Conversely, the classic presentation of T1D was easily excluded due to the preservation of robust C-peptide levels and the absolute negativity of pancreatic autoantibodies (ICA, GAD, and IAA) [3,7,8].

Distinguishing Among Monogenic Subtypes

Once an inherited form of early-onset diabetes was suspected, differentiating between specific MODY subtypes was necessary to guide management. Among MODY subtypes, HNF1A-MODY is the most prevalent and is characterized by progressive fasting hyperglycemia and osmotic symptoms (e.g., polyuria and polydipsia), with ketosis being rare due to residual insulin secretion. The second most common form, GCK-MODY, presents with mild, stable fasting hyperglycemia, is often asymptomatic, and is frequently incidentally detected. Other MODY subtypes may be suggested by their associated clinical features, such as renal abnormalities in HNF1B-MODY, or macrosomia and neonatal hypoglycemia in HNF4A-MODY [1,4,9].

Molecular mechanisms and pathogenicity of silent variants

The definitive diagnosis of HNF1A-MODY is classically established through the identification of nonsense, missense, or frameshift mutations that directly disrupt the protein structure. In this family, however, molecular analysis revealed a heterozygous synonymous (silent) variant in exon 8 (c.1533G>A; p. Gln511=).

Although bioinformatics tools and the ACMG guidelines initially classify such synonymous findings as VUS due to the lack of structural amino acid changes, a growing body of literature indicates that silent mutations can be clinically relevant [11-14]. Synonymous variants can severely impact protein expression by disrupting pre-mRNA splicing kinetics, introducing cryptic splice sites, or altering mRNA stability and translation efficiency [11,13,14]. The extreme rarity of this specific variant in global population constraint databases further supports its evolutionary intolerance and potential role in disease pathogenesis [12].

Recent functional evidence in monogenic diabetes models has demonstrated that synonymous variants can severely disrupt pre-mRNA splicing kinetics at exon boundaries, leading to downstream translational failure [13]. This aligns with the updated specifications from the ClinGen Monogenic Diabetes Expert Panel, which urge clinicians to look beyond amino acid changes and scrutinize potential splicing-disrupting variants in the HNF1A gene [15].

Incomplete Penetrance and Variable Expressivity

The clinical history of this family illustrates the complex phenomena of incomplete penetrance and variable expressivity often tied to non-classical genetic variants, a characteristic increasingly recognized in monogenic diabetes cohorts [2,7]. The mother remained entirely asymptomatic to date and lacked any medical history of hyperglycemia, despite carrying the exact same heterozygous variant. This suggests that the c.1533G>A variant may result in a partial or "leaky" molecular defect rather than a complete loss of function, rendering the clinical phenotype highly susceptible to epigenetic or environmental modifiers [2,7,8]. Such variable expressivity is also evident in the siblings, where the sister presented with obesity and mild metabolic dysfunction during puberty, while the younger brother developed acute osmotic symptoms at preschool age.

Therapeutic implications and clinical course

A precise molecular diagnosis in HNF1A-MODY carries profound therapeutic implications, given the extraordinary sensitivity of these patients to oral sulfonylureas [1,7]. Sulfonylureas close ATP-sensitive potassium channels directly, bypassing the upstream glucose-sensing defect, and remain the first-line pharmacological choice due to their high efficacy compared with insulin [7].

In this family, however, the mild nature of the genetic defect allowed both siblings to achieve sustained glycemic stability through dietary modification alone, characterized by the restriction of rapidly absorbable carbohydrates and regular physical activity. The patient has maintained an HbA1c of 5.1% over a four-year follow-up period without any pharmacological aid, a phenomenon that underscores the slow and gentle progression of this specific variant. Nonetheless, because HNF1A-MODY carries a long-term risk of microvascular complications similar to polygenic diabetes, close biannual endocrinology follow-up remains mandatory to detect early signs of β-cell decline.

## Conclusions

HNF1A-MODY should be highly suspected in cases of multi-generational diabetes where classical features of polygenic diabetes are absent, even when genetic testing reveals a VUS. The extreme rarity of the c.1533G>A variant and its strong clinical segregation within this family suggest that silent HNF1A mutations can be highly relevant, likely through non-classical pathogenic mechanisms such as altered pre-mRNA splicing or mRNA instability. This case expands our understanding of monogenic diabetes by demonstrating that synonymous variants can lead to a partial or leaky molecular defect, which clinically translates into incomplete penetrance and significant variable expressivity among family members. Ultimately, a personalized approach that combines clinical phenotyping, longitudinal tracking, and updated genetic guidelines is essential to ensure early diagnosis and avoid inappropriate therapeutic pathways in rare monogenic diabetes subtypes.
